# *De Novo* Transcriptomes of Olfactory Epithelium Reveal the Genes and Pathways for Spawning Migration in Japanese Grenadier Anchovy (*Coilia nasus*)

**DOI:** 10.1371/journal.pone.0103832

**Published:** 2014-08-01

**Authors:** Guoli Zhu, Liangjiang Wang, Wenqiao Tang, Dong Liu, Jinquan Yang

**Affiliations:** 1 College of Fisheries and Life Science, Shanghai Ocean University, Shanghai, China; 2 Department of Genetics and Biochemistry, Clemson University, Clemson, South Carolina, United States of America; Duke University, United States of America

## Abstract

**Background:**

*Coilia nasus* (Japanese grenadier anchovy) undergoes spawning migration from the ocean to fresh water inland. Previous studies have suggested that anadromous fish use olfactory cues to perform successful migration to spawn. However, limited genomic information is available for *C. nasus*. To understand the molecular mechanisms of spawning migration, it is essential to identify the genes and pathways involved in the migratory behavior of *C. nasus*.

**Results:**

Using *de novo* transcriptome sequencing and assembly, we constructed two transcriptomes of the olfactory epithelium from wild anadromous and non-anadromous *C. nasus*. Over 178 million high-quality clean reads were generated using Illumina sequencing technology and assembled into 176,510 unigenes (mean length: 843 bp). About 51% (89,456) of the unigenes were functionally annotated using protein databases. Gene ontology analysis of the transcriptomes indicated gene enrichment not only in signal detection and transduction, but also in regulation and enzymatic activity. The potential genes and pathways involved in the migratory behavior were identified. In addition, simple sequence repeats and single nucleotide polymorphisms were analyzed to identify potential molecular markers.

**Conclusion:**

We, for the first time, obtained high-quality *de novo* transcriptomes of *C. nasus* using a high-throughput sequencing approach. Our study lays the foundation for further investigation of *C. nasus* spawning migration and genome evolution.

## Introduction

The Japanese grenadier anchovy (*Coilia nasus*) is a small commercial fish in China, which belongs to the family of Engraulidae, order of Clupeiformes [1]. It is renowned for its delicate and tender meat. Moreover, *C. nasus* is well known for the long-distance ocean–river spawning migration of its anadromous population.

*C. nasus* lives in coastal ocean water for most of its lifetime, and normally reaches sexual maturity at the age of 1–2 years. *C. nasus* spawns between February and September [2]. Every year, when the spawning period arrives, thousands of mature *C. nasus* individuals undergo a long-distance migration from coastal ocean up to exorheic rivers, such as the Yangtze River, and then spawn in the lower and middle reaches of these rivers and adjacent lakes. Interestingly, the sedentary population of *C. nasus* in lakes has abandoned the long-distance migration for unknown reasons and become permanent residents there.

The ability to recognize the spawning ground is a key skill for successful reproduction. Recently, there has been a sharp decline in the population of anadromous *C. nasus* because of environmental pollution, overfishing and the destruction of spawning grounds. Therefore, the understanding of *C. nasus* spawning migration is essential for its conservation and stock management. However, little is known about the molecular basis of *C. nasus* spawning migration.

Previous studies on fish migration have mostly focused on salmonids. It has been hypothesized that salmonids use olfactory cues to return to natal rivers to spawn. Several studies, wherein the salmonid olfactory epithelium was altered, have concluded that salmonids without olfactory ability cannot discriminate natal streams and that functional olfactory ability is essential for their migration to spawn [3]–[7]. Similar conclusion was also drawn for American eels, and with the functional olfactory ability absent, anosmic eels lost the ability to migrate out of the estuary during the fall spawning migration [8]. Olfactory imprinting of dissolved amino acids in natal stream water has been reported in lacustrine sockeye salmon [9], and strong olfactory responses to natal stream water have also been found in sockeye salmon [10]. In wild anadromous Atlantic salmon, some of the olfactory receptor genes involved in the migration for reproduction have been identified [11]. These studies suggest that olfaction may be essential for the migration for reproduction in fish.

The olfactory epithelium in the nasal cavity is involved in the olfaction of fish. The olfactory functions of fish are induced by odorant elements such as steroids, bile acids and amino acids in water through the olfactory receptors in the olfactory epithelium. Subsequently, the information is processed by the central nervous system of fish to achieve the olfactory functions. To investigate the relationship between olfaction and the anadromous behavior of *C. nasus*, we sequenced the transcripts expressed in the olfactory epithelium. With this sequence information, we identified the genes and pathways involved in the migratory behavior of *C. nasus*. At present, little genomic information about *C. nasus* is available in the National Center for Biotechnology Information (NCBI) database. Therefore, the high-quality transcriptome data obtained in this study will be useful for future research on *C. nasus*.

## Results and Discussion

### Transcriptome sequencing and assembly

As described in the Materials and Methods, cDNA libraries for the olfactory sac of wild anadromous and non-anadromous *C. nasus* were constructed and sequenced using the Illumina platform, which produced 51,261,228 and 126,241,752 clean reads, respectively (Table 1). For anadromous and non-anadromous *C. nasus*, 117,717 and 231,219 unigenes, respectively, were obtained, and 176,510 unigenes with a mean length of 843 nucleotides were assembled from the anadromous and non-anadromous *C. nasus* unigenes (Table 1 and Figure S1). The total length of the 176,510 assembled unigenes was 148,772,175 nucleotides.

**Table 1:** Summary of the sequences obtained from the olfactory epithelium of anadromous and non-anadromous *Coilia nasus*.

	Anadromous	Non-anadromous	
Total clean reads	51,261,228	126,241,752	
Total clean nucleotides (nt)	4,613,510,520	12,750,416,952	
Contig total number	223,325	409,459	
Unigene total number	117,717	231,219	
Contig total length (nt)	56,758,068	129,299,285	
Unigene total length (nt)	50,868,550	197,568,883	
All total number			176,510
Alltotal length (nt)			148,772,175

The quality of the sequence assembly result and the size distribution are shown in Figure S1. Of all the unigenes, 8,608 or over 4.8% are ≥3,000 nucleotides in length. The coding regions have been identified for 81,315 sequences (72,601 using BLASTX and 8,714 using expressed sequence tag scan; Figure S2). While it is time-consuming to obtain large cDNA collections using the traditional Sanger sequencing method, the next-generation sequencing platform has been demonstrated in this study to be useful for efficiently generating high-quality transcriptome data of *C. nasus*.

### Annotation of predicted proteins and classification using COG

The putative functions of 89,456 unigenes (50.68% of all unigenes) were annotated by sequence similarity analysis with E value ≤1×10^−5^ (72,127 using the NR database, 65,888 using the NT database, 61,581 using the SwissProt database, 53,575 using the KEGG database, 25,272 using the COG database, and 41,888 using gene ontology terms). However, because of the lack of genome and EST sequence data from *C. nasus*, approximately 49.32% of the unigenes could not be functionally annotated.

The E-value distribution and similarity distribution for the 72,127 unigenes (40.86% of all unigenes) that were annotated using the NR database are shown in Figure S3. The species distribution of the best BLASTX hits is also shown in Figure S3. About 66.2% of the unigenes were functionally annotated with the known fish genes. However, a small number of sequences were matched to *Paramecium tetraurelia* and *Tetrahymena thermophila* SB210 genes. These sequences may represent contaminants from sample collection or parasitic infection of *C. nasus*.

**Figure 1:**
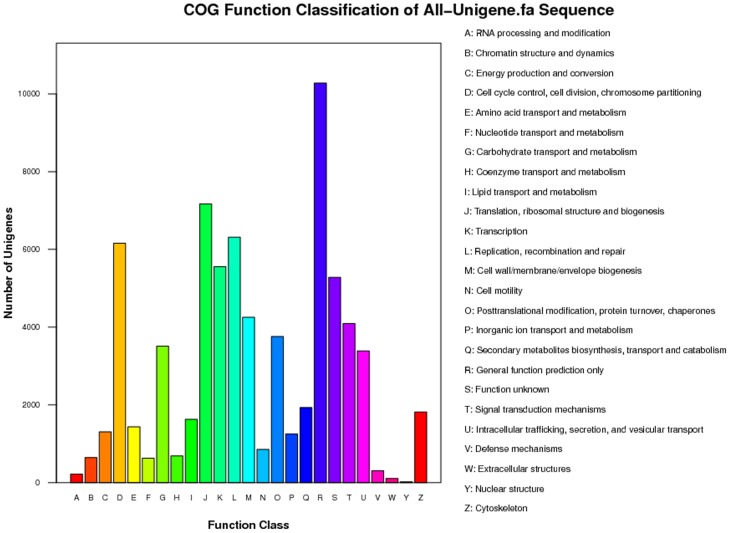
Histogram presentation of the results from the classification using the Clusters of Orthologous Groups (COG).

COG (clusters of orthologous groups of proteins) is a database where orthologous gene products are classified into different clusters. A total of 25,272 *C. nasus* unigenes were assigned to 25 COG categories with E value ≤1×10^−5^ (Figure 1). Among these COG categories, the cluster for “general function prediction” was the largest, containing 10,278 (40.66%) of the unigenes, followed by “translation, ribosomal structure, and biogenesis” (7,169 or 28.36%), “replication, recombination, and repair” (6,315 or 24.98%), and “cell cycle control, cell division, chromosome partitioning” (6,161 or 24.37%). In addition, the “signal transduction mechanisms” cluster contained 4,092 (16.19%) unigenes.

### Gene ontology assignments

**Figure 2:**
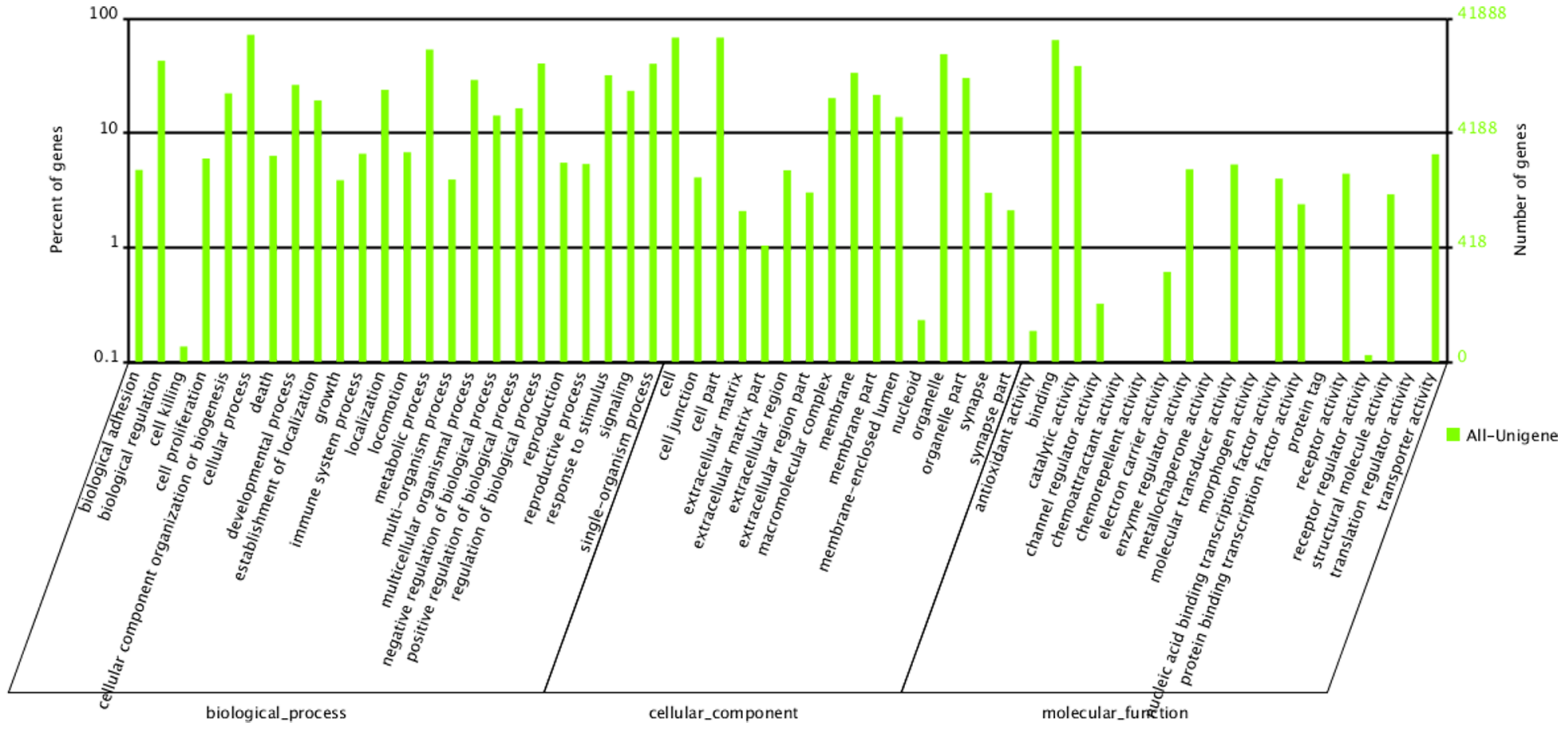
Histogram presentation of Gene Ontology (GO) classification. The results are divided into three GO categories: biological processes, cellular components, and molecular functions.

To understand the functional capacity of the *C. nasus* transcriptome, 41,888 unigenes (46.8% of all unigenes) were assigned to three Gene Ontology (GO) categories: biological processes, cellular components and molecular functions (Figure 2). In the GO category of biological processes, 13,391 unigenes were involved in response to stimulus and 9,782 in signaling, both of which were enriched in this category. Of the unigenes assigned to the GO category of cellular components, 9,021 were involved in the membrane part. In addition, of the unigenes annotated with potential molecular functions, binding (27,140) and catalytic activity (16,082) were enriched in this category. GO terms of channel regulator activity (135 unigenes), electron carrier activity (256), receptor activity (1,845), and receptor regulator activity (48) were also well represented. The large number of regulatory transcripts found in our data may indicate transcriptional plasticity in the olfactory epithelium.

Approximately 41.9% of all the transcripts of *C. nasus* did not have GO terms assigned to them. This may be because of the fact that knowledge regarding the function of *C. nasus* genes is currently limited. It is also possible that these transcripts are from non-coding RNA genes. Nevertheless, the unannotated transcripts in the olfactory epithelium should be documented as they may be involved in the olfaction of *C. nasus*, either directly or indirectly.

Previous studies on the transcriptome of fish olfactory epithelium have been limited to the goldfish *Carassius auratus* [12]. Since this goldfish does not have the ability to migrate, comparing *C. auratus* and *C. nasus* transcriptomes may provide useful information on the molecular mechanisms of migration. We compared the GO terms of response to stimulus and binding, which may be involved in olfaction and signal transduction. *C. nasus* had a higher proportion of both terms than *C. auratus* (6.30% versus 4.40% in response to stimulus; 47.90% versus 45.70% in binding), suggesting that *C. nasus* may have higher olfaction ability than *C. auratus*.

### Kyoto Encyclopedia of Genes and Genomes (KEGG) analysis

A total of 53,575 unigenes were annotated with the genes in the KEGG database. The number of unigenes in different pathways ranged from 2 to 5,243. The top 25 pathways with the highest sequence tag numbers are shown in Table 2. The top pathway (metabolic pathway) contained 5,243 unigenes. These predicted KEGG pathways may provide a useful resource for research into the spawning migration of *C. nasus* and other molecular studies in *C. nasus*.

**Table 2:** List of the top 25*Coilia nasus* transcriptomes.

No.	Pathway	Number (%) of ESTs	Pathway ID
1	Metabolic pathways	5,243 (9.79)	ko01100
2	Regulation of actin cytoskeleton	2,772 (5.17)	ko04810
3	Pathways in cancer	2,671 (4.99)	ko05200
4	Amoebiasis	2,288 (4.27)	ko05146
5	Focal adhesion	2,274 (4.24)	ko04510
6	Spliceosome	2,226 (4.15)	ko03040
7	MAPK signaling pathway	1,758 (3.28)	ko04010
8	RNA transport	1,651 (3.08)	ko03013
9	Endocytosis	1,602 (2.99)	ko04144
10	Tight junction	1,596 (2.98)	ko04530
11	Huntington’s disease	1,581 (2.95)	ko05016
12	HTLV-I infection	1,578 (2.95)	ko05166
13	Salmonella infection	1,570 (2.93)	ko05132
14	Herpes simplex infection	1,491 (2.78)	ko05168
15	Adherens junction	1,458 (2.72)	ko04520
16	Influenza A	1,443 (2.69)	ko05164
17	Chemokine signaling pathway	1,437 (2.68)	ko04062
18	Vibrio cholerae infection	1,436 (2.68)	ko05110
19	Epstein-Barr virus infection	1,427 (2.66)	ko05169
20	Fc gamma R-mediated phagocytosis	1,378 (2.57)	ko04666
21	Vascular smooth muscle contraction	1,352 (2.52)	ko04270
22	Dilated cardiomyopathy	1,327 (2.48)	ko05414
23	Hypertrophic cardiomyopathy (HCM)	1,261 (2.35)	ko05410
24	Calcium signaling pathway	1,251 (2.34)	ko04020
25	Transcriptional misregulation in cancer	1,240 (2.31)	ko05202

### Simple sequence repeats (SSRs) and SNPs as genetic markers

Molecular markers are a useful tool for species evolution and population differentiation studies. At present, studies of the *C. nasus* population are restricted by the lack of effective molecular markers. Through *de novo* assembly of transcriptome data, 78,852 SSRs in 54,059 sequences were detected. These SSRs include 14,998 monomers, 50,071 dimers, 9,546 trimers, 2,317 quadmers, 1,523 pentamers, and 397 hexamers (Figure S4). In addition, 224,779 single nucleotide polymorphism (SNP) sites were identified. 93,501 sites were found in anadromous *C. nasus* and 131,278 in non-anadromous *C. nasus*. There were 138,945 transition sites and 85,734 transversion sites (Table S1). The large number of putative molecular markers identified in our work may be useful for future studies on the evolution of the *C. nasus* genome, such as gene flow, genetic mapping, and genotyping.

### A resource for investigation of migration genes

Previous studies on the migration of *C. nasus* have mainly focused on the behavioral and morphology aspects [1], [2], [13]–[19]. In this study, we aimed to expand this knowledge and provide new insight into the molecular mechanism of *C. nasus* migration. The transcriptome data obtained in this study provide a good resource for identifying the putative genes involved in *C. nasus* migration.

#### Pathway of olfactory transduction

The hypothesis of olfactory imprinting and homing for salmon assumes that some odorant molecules in the natal stream are imprinted on the olfactory system of juvenile salmon during their downstream migration, and adult salmon detect the corresponding molecules to discriminate the natal stream during their homing migration [9], [10], [20].

**Figure 3:**
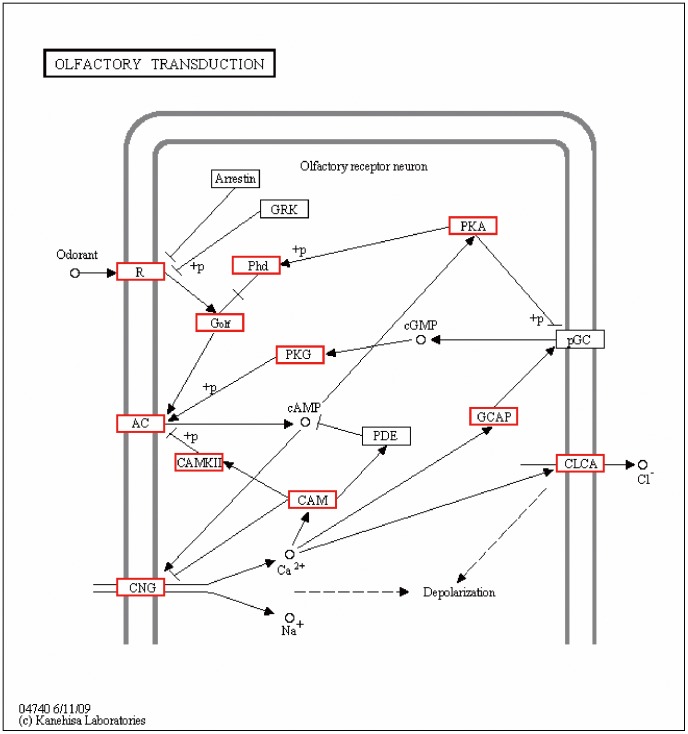
Functional annotation of *Coilia nasus* genes using the KEGG pathway of olfactory transduction. The genes identified in the *C. nasus* transcriptomes are shown in red boxes. R: odorant receptor; G_olf_: G_αolf_-containing heterotrimeric G protein; AC: adenylate cyclase; CNG: cyclic nucleotide-gated cation channel; CLCA: calcium-activated chloride channel; GCAP: guanylyl cyclase-activating protein; Phd: phosducin; PKG: cGMP-dependent protein kinase; PKA: protein kinase A; pGC: particulate guanylyl cyclase; CAM: calmodulin; CAMKII: calcium/calmodulin-dependent protein kinase (CaM kinase) II; PDE: phosphodiesterase; Arrestin: arrestin; GRK: G protein receptor kinase.

In our study, the KEGG pathway of olfactory transduction (ko04740) [21]–[29] was used to annotate the largest number of genes (Figure 3). 547 unigenes, or 1.02% of the KEGG-annotated unigenes, were assigned to the olfactory transduction pathway.

At present, little is known about the pathway of olfactory transduction in *C. nasus*; however, relevant information can be obtained from other vertebrate species [30]. The canonical pathway of the olfactory transduction is initiated from the detection of odor molecules by odorant receptors (Rs). Binding of the odor molecules to the odorant receptors activates the Gα_olf_-containing heterotrimeric G protein (G_olf_), which then activates adenylyl cyclase (AC) to produce cAMP [31]. Subsequently, cAMP opens the cyclic nucleotide-gated cation channels (CNG) [32]. Ca^2+^ ions influx into the cells and depolarization occurs. Ca^2+^-activated chloride channels (CLCA) allow an efflux of Cl^−^ ions, which leads to further depolarization of the cell [33]–[38]. The chemical signals are then converted into electronic signals that are delivered to the brain, where the signals are perceived as smells.

Elevated intracellular Ca^2+^ triggers multiple molecular events, including the down-regulation of the affinity of the CNG channel to cAMP and inhibition of the activity of AC via CAMKII (calcium/calmodulin-dependent protein kinase II)-dependent phosphorylation [24]. Longer exposure to odorants can stimulate particulate guanylyl cyclase (pGC) in cilia to produce cGMP and activate cGMP-dependent protein kinase (PKG), leading to a further increase in the amount and duration of intracellular cAMP levels, which may function to convert inactive forms of protein kinase A (PKA) to active forms [39]. PKA can also inhibit the activation of pGC as a feedback.

Termination of the response may occur at all steps of the pathway, which include receptor phosphorylation by G protein receptor kinase (GRK) or protein kinase A (PKA) and ‘capping’ of the phosphorylated receptor by arrestin [40]–[42], inhibition of adenylyl cyclase activity by CaMKII and regulation of G protein signaling 2 (RGS2) [43], [44], removal of Ca^2+^ through a Na^+^–Ca^2+^ exchanger [45], hydrolysis of cAMP by phosphodiesterase (PDE) activity, and desensitization of the CNG channel by Ca^2+^-calmodulin (CAM)-dependent processes [46]. However, the transcripts of arrestin, GRK and PDE involved in the response termination, and pGC are not detected in this study. This may be because *C. nasus* has a unique pathway with a lower termination ability. Since several terminators are absent in the olfactory transduction, sustained detection of odor elements in natal rivers may be possible for *C. nasus*. It is also possible that these transcripts are rare and thus undetected in this study.

#### Putative pheromone signaling pathway

The pheromone hypothesis was proposed based on research on Atlantic salmon *Salmon salar* and Arctic char *Salvelinus alpines* [47]. In sea lamprey, a mixture of sulfated steroids has also been demonstrated to function as a migratory pheromone [48]. Thus, the putative pheromone signaling pathway should also be considered in the study of the migration behavior of *C. nasus*.

Pheromones are secreted or excreted chemicals that can impact on the behavior of a receiving individual and trigger a social response within members of the same species. Vomeronasal type-1 receptors (V1Rs) and vomeronasal type-2 receptors (V2Rs) have been shown to function as pheromone receptors [49], [50]. The binding of a pheromone to a V1R activates inhibitory adenylate cyclase G protein (Gi), and phospholipase Cβ2 (PLCβ2) is activated to produce inositol-1,4,5-trisphoshate and diacylglycerol from phosphatidylinositol-4,5-bisphoshate. This activates the transient receptor potential cation channel C2 (TRPC2). Activation of TRPC2 allows a Na^+^/Ca^2+^ influx, which leads to depolarization. Recovery and adaptation of response may involve binding of CaM to TRPC2. The binding of pheromones to V2Rs activates G_o_, which is a G protein involved in many signal transduction channels [30]. In V2R-expressing neurons, TRPC2 has been shown to generate depolarizing currents [30]. In this study, we identified the family of V1R and V2R, and CaM in the transcriptomes of *C. nasus*. However, TRPC2 was not detected although we identified the other members of transient receptor potential cation channels, including TRPM4, TRPV4, TRPC5, and TRPV1. It is possible that the role of TRPC2 in the pheromone signaling pathway may be superseded by the other members of the gene family.

## Conclusion

By using a high-throughput sequencing approach, we obtained the high-quality *de novo* transcriptomes of *C. nasus* for the first time. Our data provide valuable information for understanding the spawning migration of *C. nasus*, and lay the foundation for future research on the genome evolution of this species, especially as the genomic sequence is still unavailable for *C. nasus*.

## Materials and Methods

### Ethics statement

The study was approved by the Institutional Animal Care and Use Committee of Shanghai Ocean University and performed in strict accordance with the Guidelines on the Care and Use of Animals for Scientific Purposes set by the Institutional Animal Care and Use Committee of Shanghai Ocean University.

### Fish material

Three males of non-anadromous *C. nasus* were collected from Poyang Lake in Jiujiang, Jiangxi Province in China at the end of March 2012 when anadromous males of *C. nasus* had not reached Poyang Lake to spawn. The fish collection was performed with the help of fisherman Baishan Zhan with the fishing license (No. 0400051) permitted by the Jiangxi Provincial Department of Agriculture. One male of anadromous *C. nasus* was collected from the Jingjiang section of the Yangtze River in Jingjiang, Jiangsu Province in China at the beginning of April 2012 when they were migrating to spawning grounds along the Yangtze River. The fish collection was performed with the assistance of fisherman Xiping Zhou with the fishing license (No. SuChuanBu 2011 JMF254) and the special fishing license of C. nasus in the Yangtze River (No. SuChuanBu 2012 ZX-M032) permitted by Jiangsu Provincial Oceanic and Fishery Bureau. All fish collections were carried out in wild water, and the captured live C. nasus was immediately buried in medical ice bags (−20°C) until the loss of consciousness.

Before sampling, the *C. nasus* was dissected on ice and subsequently the anatomical characters of the testis gonadal development phase of *C. nasus* were rapidly checked [51]. If the individual’s testis gonadal development phase was in phase III, then the olfactory capsules of *C. nasus* were collected. The operations were completed within 10 min after the loss of consciousness. After this procedure, the olfactory capsules from the non-anadromous *C. nasus* were placed into 2.0 mL tubes containing RNAlater (Ambion, US). Then the collected olfactory samples were stored at 4°C overnight and stored at −20°C for 12 hours during the delivery to Shanghai Ocean University, where the samples were transferred to −80°C before processing. The olfactory capsules from the anadromous *C. nasus* were immediately placed into 2.0 mL tubes and frozen in liquid nitrogen after collection and then delivered to the Shanghai Ocean University for further processing. All the remains of above sampled fish were stored in freezer.

### RNA extraction

Total RNA was isolated from samples using TRIzol reagent (Invitrogen, USA) according to the manufacturer’s instructions. The quality of purified RNA was verified on a 2100-Bioanalyzer (Agilent, USA). To prevent DNA contamination, the RNA samples were treated with DNase I. The high-quality RNA samples were then used for further experiments.

### cDNA preparation and library construction

Poly(A)-containing mRNA samples were captured from total RNA with Oligo (dT)-Bead complex. The fragment mixture of the RNA fragmentation kit was added to mRNA to obtain RNA pieces with different lengths. Then single- and double-stranded cDNAs were synthesized from mRNA samples through reverse transcription using high-quality total RNA as the starting material.

The following cDNA purification was then performed. Purified cDNA fragments were suspended into End Repair Mix for end reparation and adenylate 3′ ends. Short fragments produced from the above procedures were ligated with sequencing adaptors, and then fragments with adaptors were purified and enriched with cDNA fragments through PCR. Subsequently, the purified PCR products were used to create a cDNA library. The size distribution and accurate quantification of the library were checked on a 2100-Bioanalyzer (Agilent, USA) and an ABI StepOnePlus Real-Time PCR System.

### cDNA library sequencing

cDNA libraries were constructed for sequencing with Illumina Hiseq 2000. Raw sequence data were processed through the trimming of adaptor sequences, ambiguous nucleotides, and empty reads to obtain the clean data. With software Trinity and TIGR Gene Indices (TGI) Clustering tools v2.1 [52], [53], the short clean reads obtained from the two types of *C. nasus* were assembled and clustered. Sequences with the fewest nucleotides that could not be extended on either end were then obtained. These sequences were called unigenes.

### Unigene functional annotation and classification

The unigenes were functionally annotated by searching databases, including NR (ftp://ftp.ncbi.nih.gov/blast/db/), NT (ftp://ftp.ncbi.nih.gov/blast/db/), SwissProt (ftp://ftp.uniprot.org/pub/databases/uniprot/previous_releases/), COG (http://www.ncbi.nlm.nih.gov/COG/), gene ontology (http://www.geneontology.org/) and KEEG (http://www.genome.jp/), using BLAST with E-value ≤1×10^−5^. The ESTSscan software v3.0.2 (http://www.ch.embnet.org/software/ESTScan2.html) was used to predict the coding region if a unigene had not been annotated using one of the previously mentioned databases.

Functional annotation using Gene Ontology terms (molecular functions, cellular components, and biological processes) was performed using BLAST2GO software v2.5.0 based on the NR annotation information [54]. After the gene ontology annotation, WEGO was used to obtain Gene Ontology function classification statistics of all the unigenes for understanding the species’ gene function distribution [55].

The Kyoto Encyclopedia of Genes and Genomes (KEGG) database provides a systematic analysis of metabolic pathways and functions of gene products. In this study, the *C. nasus* unigenes were assigned to canonical pathways described in KEGG using BLASTX.

### SSRs and SNPs analysis

Simple sequence repeats (SSRs) in the *C. nasus* unigenes were detected using the microsatellite identification tool (MISA) (http://pgrc.ipk-gatersleben.de/misa/). Detection criteria of SSRs included perfect repeat motifs of one to six base pairs and a minimum repeat number of 12 for mono-, six for di-, five for tri-, five for tetra-, four for penta-, and four for hexa-nucleotide microsatellites. SOAPsnp (http://soap.genomics.org.cn/soapsnp.html) was used to detect single nucleotide polymorphisms (SNPs) in the *C. nasus* unigenes.

### Data deposition

The raw Illumina sequencing data from the olfactory epithelium of *C. nasus* were deposited in the NCBI Sequence Read Archive (SRA) Sequence Database (accession number SRP035517).

## Supporting Information

Figure S1 **The length distribution of all unigenes.**
(TIF)

Figure S2 **The distribution of coding sequence region of all unigenes obtained by BLASTX and EST scan.**
(TIF)

Figure S3 **The NR database classification.**
(TIF)

Figure S4 **Simple sequence repeat statistics.**
(TIF)

Table S1 **Single nucleotide polymorphism statistics.**
(XLSX)

## References

[1] Whitehead PJP, Nelson GJ, Wongratana T (1988) FAO species catalogue. Clupeoid fishes of the world (Suborder Clupeoidei). Part 2. Engraulididae. FAO Fisheries Synopsis 125(7): 460–475.

[2] Yuan CM, Qin AL, Liu RH (1980) Discussion on subspecific taxonomy of the genus *Coilia* in middle and lower reaches of Yangtze River and southeast coastal China Sea. Journal of Nanjing University (Natural Sciences) 3: 67–82.

[3] McBride JR, Fagerlund UHM, Smith M, Tomlinson N (1964) Olfactory preception in juvenile salmon: II. Conditioned response of juvenile sockeye salmon (*Oncorhynchus neeka*) to lake waters. Canadian Journal of Zoology 42(2): 245–248.

[4] Tarrant RM (1966) Threshold of perception of eugenol in juvenile sockeye salmon. Transactions of the American Fisheries Society 95(1): 112–115.

[5] Jahn LA (1967) Responses to odors by fingerling cutthroat trout from Yellowstone Lake. The Progressive Fish-Culturist 38(4): 207–210.

[6] Doving KB, Westerberg H, Johnsen PB (1985) Role of olfaction in the behavior and neural responses of Atlantic salmon, *Salmon salar*, to hydrographic stratification. Canadian Journal of Fisheries and Aquatic Sciences 42(10): 1658–1667.

[7] Yano K, Nakamura A (1992) Observations on the effect of visual and olfactory ablation on the swimming behavior of migrating adult chum salmon, *Oncorhynchus keta* . Ichthyological Research 39(1): 67–83.

[8] Barbin GP, Parher SJ, McCleave JD (1998) Olfactory clues play a critical role in the estuarine migration of silver-phase American eels. Environmental Biology of Fishes 53: 283–291.

[9] Yamamoto Y, Hino H, Ueda H (2010) Olfactory Imprinting of Amino Acids in n Lacustrine Sockeye Salmon. PLoS ONE 5(1): e8633. 20062811 10.1371/journal.pone.0008633 PMC2799659

[10] Bandoh H, Kida I, Ueda H (2011) Olfactory Responses to Natal Stream Water in Sockeye Salmon by BOLD fMRI. PLoS ONE 6(1): e16051. 21264223 10.1371/journal.pone.0016051 PMC3022028

[11] Johnstone KA, Lubieniecki KP, Koop BF, Davidson WS (2011) Expression of olfactory receptors in different life stages and life histories of wild Atlantic salmon (*Salmo salar*). Molecular Ecology 20(19): 4059–4069. 21883590 10.1111/j.1365-294X.2011.05251.x

[12] Kolmakov NN, Kube MK, Reinhardt R, Canario AV (2008) Analysis of the goldfish *Carassius auratus* olfactory epithelium transcriptome reveals the presence of numerous non-olfactory GPCR and putative receptors for progestin pheromones. BMC genomics 9: 429–445. 18803863 10.1186/1471-2164-9-429 PMC2556351

[13] Yang JQ, Hu XL, Tang WQ, Lin HD (2008) mtDNA control region sequence variation and genetic diversity of *Coilia nasus* in Yangtze River estuary and its adjacent waters. Chinese Journal of Zoology 43(1): 8–15.

[14] Guo HY, Tang WQ (2006) The relationship between sagittal otolith weight-age and its use in age determination in *Coilia nasus* from the estuary of Yangtze River. Journal of Fisheries of China 30(3): 347–352.

[15] Guan WB, Chen HH, Ding HT, Xuan FJ, Dai XJ (2010) Reproductive characteristics and conditions of anadromous *Coilia ectenes* (Engraulidae) in Yangtze estuary. Marine Fisheries 32(1): 73–81.

[16] Cheng WX, Tang WQ (2011) Some phenotypic varieties between different ecotypes of *Coilia nasus* in Yangtze River. Chinese Journal of Zoology 46: 33–40.

[17] Zheng F, Guo HY, Tang WQ, Li HH, Liu D, et al (2012) Age structure and growth characteristics of anadromous populations of *Coilia nasus* in the Yangtze River. Chinese Journal of Zoology 47(5): 24–31.

[18] Xu G, Wan J, Gu R, Zhang C, Xu P (2013) Morphological and histological studies on ovary development of *Coilia nasus* under artificial farming conditions. Journal of Fishery Sciences of China 18: 537–546.

[19] Xu GC, Dong JJ, Nie ZJ, Xu P, Gu RB (2012) Studies on lactate dehydrogenase isozymes and DNA content in different tissues of *Coilia nasus* . Journal of Shanghai Ocean University 21(4): 481–488.

[20] Wisby WJ, Hasler AD (1954) Effect of olfactory occlusion on migrating silver salmon (*O. kisutch*). Journal of the Fisheries Board of Canada 11(4): 472–478.

[21] Firestein S (2001) How the olfactory system makes sense of scents. Nature 413(6852): 211–218. 11557990 10.1038/35093026

[22] Ache BW, Young JM (2005) Olfaction: diverse species, conserved principles. Neuron 48(3): 417–430. 16269360 10.1016/j.neuron.2005.10.022

[23] Nakamura T (2000) Cellular and molecular constituents of olfactory sensation in vertebrates. Comparative Biochemistry and Physiology Part A: Molecular & Integrative Physiology 126(1): 17–32. 10.1016/s1095-6433(00)00191-4 10908849

[24] Zufall F, Leinders-Zufall T (2000) The cellular and molecular basis of odor adaptation. Chemical senses 25(4): 473–481. 10944513 10.1093/chemse/25.4.473

[25] Moon C, Jaberi P, Otto-Bruc A, Baehr W, Palczewski K, et al (1998) Calcium-sensitive particulate guanylyl cyclase as a modulator of cAMP in olfactory receptor neurons. The Journal of neuroscience 18(9): 3195–3205. 9547228 10.1523/JNEUROSCI.18-09-03195.1998 PMC6792646

[26] Ronnett GV, Moon C (2002) G proteins and olfactory signal transduction. Annual review of physiology 64(1): 189–222. 10.1146/annurev.physiol.64.082701.102219 11826268

[27] Barry PH (2003) The relative contributions of cAMP and InsP3 pathways to olfactory responses in vertebrate olfactory receptor neurons and the specificity of odorants for both pathways. The Journal of general physiology 122(3): 247–250. 12939389 10.1085/jgp.200308910 PMC2234488

[28] Kohout TA, Lefkowitz RG (2003) Regulation of G protein-coupled receptor kinases and arrestins during receptor desensitization. Molecular pharmacology 63(1): 9–18. 12488531 10.1124/mol.63.1.9

[29] Boekhoff I, Touhara K, Danner S, Inglese J, Lohse MJ, et al (1997) Phosducin, potential role in modulation of olfactory signaling. Journal of Biological Chemistry 272(7): 4606–4612. 9020189 10.1074/jbc.272.7.4606

[30] Kaupp UB (2010) Olfactory signalling in vertebrates and insects: differences andcommonalities. Nature Reviews Neuroscience 11(3): 188–200. 20145624 10.1038/nrn2789

[31] Breer H, Boekhoff I, Tareilus E (1990) Rapid kinetics of second messenger formation in olfactory transduction. Nature 345: 65–68. 2158631 10.1038/345065a0

[32] Nakamura T, Gold GH (1987) A cyclic nucleotide-gated conductance in olfactory receptor cilia. Nature 325: 442–444. 3027574 10.1038/325442a0

[33] Kleene SJ, Gesteland RC (1991) Calcium-activated chloride conductance in frog olfactory cilia. The Journal of neuroscience 11(11): 3624–3629. 1941099 10.1523/JNEUROSCI.11-11-03624.1991 PMC6575529

[34] Lowe G, Gold GH (1993) Non linear amplification by calcium-dependent chloride channels in olfactory receptor cells. Nature 366: 283–286. 8232590 10.1038/366283a0

[35] Kaneko H, Putzier I, Frings S, Kaupp UB, Gensch T (2004) Chloride accumulation in mammalian olfactory sensory neurons. The Journal of neuroscience 24(36): 7931–7938. 15356206 10.1523/JNEUROSCI.2115-04.2004 PMC6729923

[36] Reisert J, Lai J, Yau KW, Bradley J (2005) Mechanism of the excitatory Cl response in mouse olfactory receptor neurons. Neuron 45(4): 553–561. 15721241 10.1016/j.neuron.2005.01.012 PMC2877386

[37] Nickell WT, Kleene NK, Kleene SJ (2007) Mechanisms of neuronal chloride accumulation in intact mouse olfactory epithelium. The Journal of physiology 583(3): 1005–1020. 17656441 10.1113/jphysiol.2007.129601 PMC2277205

[38] Kurahashi T, Yau KW (1993) Co-existence of cationic and chloride components in odorant-induced current of vertebrate olfactory receptor cells. Nature 363: 71–74. 7683113 10.1038/363071a0

[39] Moon C, Jaberi P, Otto-Bruc A, Baehr W, Palczewski K, et al (1998) Calcium-sensitive particulate guanylyl cyclase as a modulator of cAMP in olfactory receptor neurons. The Journal of neuroscience 18(9): 3195–3205. 9547228 10.1523/JNEUROSCI.18-09-03195.1998 PMC6792646

[40] Dawson TM, Arriza JL, Jaworsky DE, Borisy FF, Attramadal H, et al (1993) Beta-adrenergic receptor kinase-2 and beta-arrestin-2 as mediators of odorant-induced desensitization. Science 259(5096): 825–829. 8381559 10.1126/science.8381559

[41] Peppel K, Boekhoff I, McDonald P, Breer H, Caron MG, et al (1997) G protein-coupled receptor kinase 3 (GRK3) gene disruption leads to loss of odorant receptor desensitization. Journal of Biological Chemistry 272(41): 25425–25428. 9325250 10.1074/jbc.272.41.25425

[42] Mashukova A, Spehr M, Hatt H, Neuhaus EM (2006) β-arrestin2-mediated internalization of mammalian odorant receptors. The Journal of neuroscience 26(39): 9902–9912. 17005854 10.1523/JNEUROSCI.2897-06.2006 PMC6674477

[43] Wei J, Zhao AZ, Chan GC, Baker LP, Impey S, et al (1998) Phosphorylation and inhibition of olfactory adenylyl cyclase by CaM kinase II in neurons: a mechanism for attenuation of olfactory signals. Neuron 21(3): 495–504. 9768837 10.1016/s0896-6273(00)80561-9

[44] Sinnarajah S, Dessauer CW, Srikumar D, Chen J, Yuen J, et al (2001) RGS2 regulates signal transduction in olfactory neurons by attenuating activation of adenylyl cyclase III. Nature 409 (6823): 1051–1055. 10.1038/35059104 11234015

[45] Reisert J, Matthews HR (1998) Na^+^-dependent Ca^2+^ extrusion governs response recovery in frog olfactory receptor cells. The Journal of general physiology 112(5): 529–535. 9806962 10.1085/jgp.112.5.529 PMC2229439

[46] Chen TY, Yau KW (1994) Direct modulation by Ca^2+^–calmodulin of cyclic nucleotide-activated channel of rat olfactory receptor neurons. Nature 368(6471): 545–548. 7511217 10.1038/368545a0

[47] Nordeng H (1971) Is the local orientation of anadromous fishes determined by pheromones? Nature 233: 411–413. 16063406 10.1038/233411a0

[48] Sorensen PW, Fine JM, Dvornikovs V, Jeffrey CS, Shao F, et al (2005) Mixture of new sulfated steroids functions as a migratory pheromone in the sea lamprey. Nature Chemical Biology 1(6): 324–328. 16408070 10.1038/nchembio739

[49] Boschat C, Pélofi C, Randin O, Roppolo D, Lüscher C, et al (2002) Pheromone detection mediated by a V1r vomeronasal receptor. Nature Neuroscience 5(12): 1261–1262. 12436115 10.1038/nn978

[50] Ryba NJP, Tirindelli R (1997) A new multigene family of putative pheromone receptors. Neuron 19(2): 371–379. 9292726 10.1016/s0896-6273(00)80946-0

[51] Xu GC, Nie ZJ, Zhang CX, Wei GL, Xu P, et al (2012) Histological studies on testis development of *Coilia nasus* under artificial farming conditions. Journal of huazhong agriculture university 31(2): 247–252.

[52] Grabherr MG, Haas BJ, YassourM, Levin JZ, Thompson DA, et al (2011) Full-length transcriptome assembly from RNA-Seq data without a reference genome. Nature biotechnology 29(7): 644–652. 10.1038/nbt.1883 PMC3571712 21572440

[53] Pertea G, Huang X, Liang F, Antonescu V, Sultana R, et al (2003) TIGR Gene Indices clustering tools (TGICL): a software system for fast clustering of large EST datasets. Bioinformatics 19(5): 651–652. 12651724 10.1093/bioinformatics/btg034

[54] Conesa A, Götz S, García-Gómez JM, Terol J, Talón M, et al (2005) Blast2GO: a universal tool for annotation, visualization and analysis in functional genomics research. Bioinformatics 21(18): 3674–3676. 16081474 10.1093/bioinformatics/bti610

[55] Ye J, Fang L, Zheng H, Zhang Y, Chen J, et al. (2006) WEGO: a web tool for plotting GO annotations. Nucleic Acids Research (suppl 2): W293–297. 10.1093/nar/gkl031 PMC1538768 16845012

