# Supplementary figures and images for: De Novo Transcriptomes of Olfactory Epithelium Reveal the Genes and Pathways for Spawning Migration in Japanese Grenadier Anchovy (Coilia nasus)
Source: PLoS One. 2014 Aug 1;9(8):e103832. doi: 10.1371/journal.pone.0103832 (PMC4118956; doi:10.1371/journal.pone.0103832)

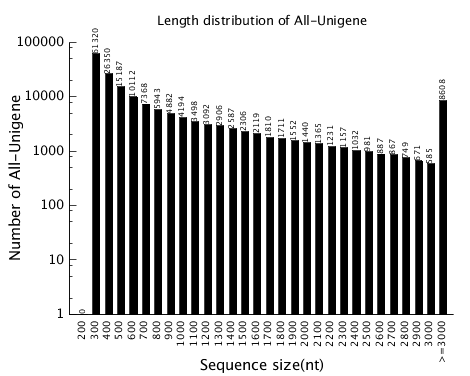

Supplement: Figure S1 — The length distribution of all unigenes. (TIF) [file pone.0103832.s001.tif]

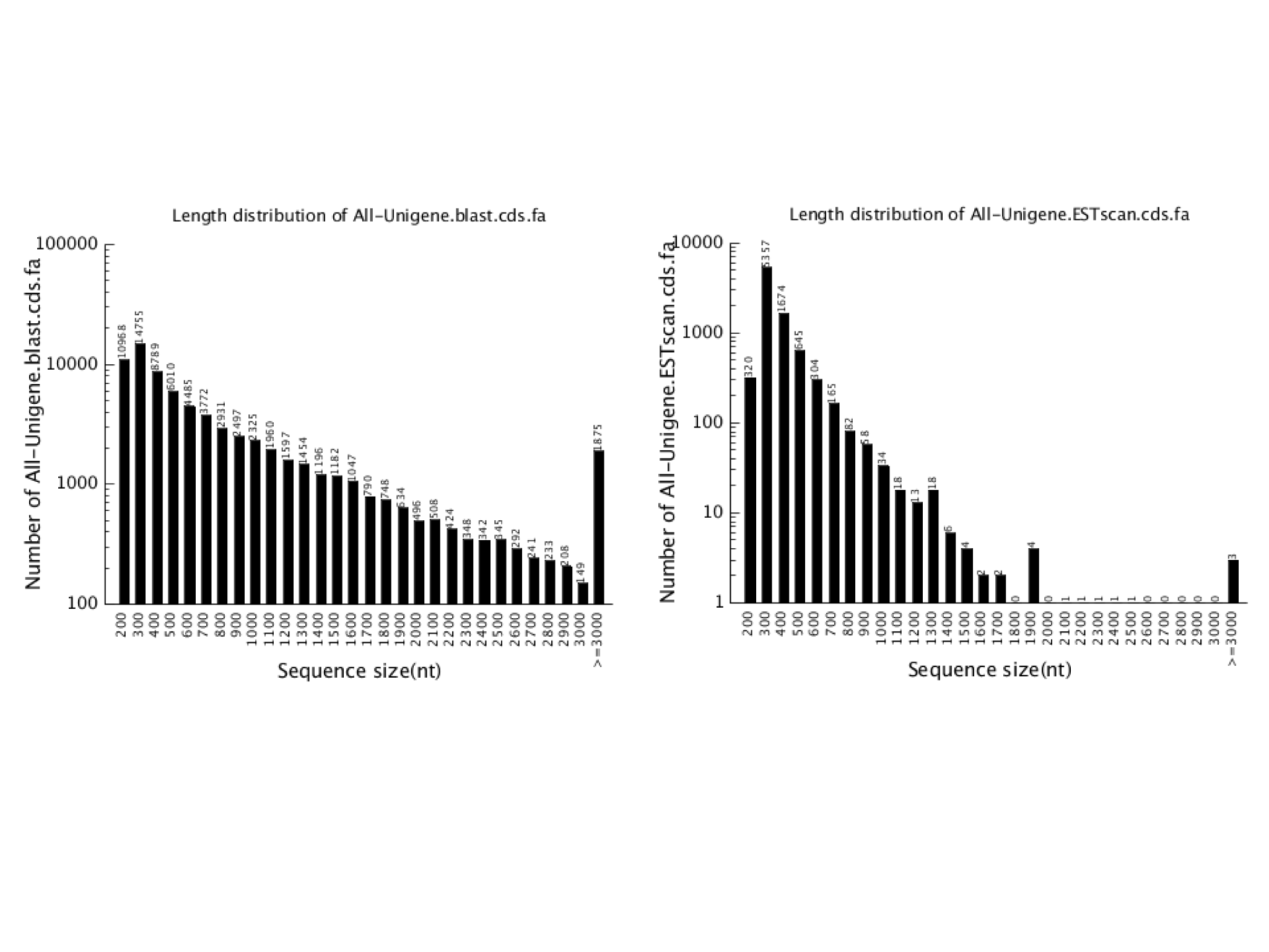

Supplement: Figure S2 — The distribution of coding sequence region of all unigenes obtained by BLASTX and EST scan. (TIF) [file pone.0103832.s002.tif]

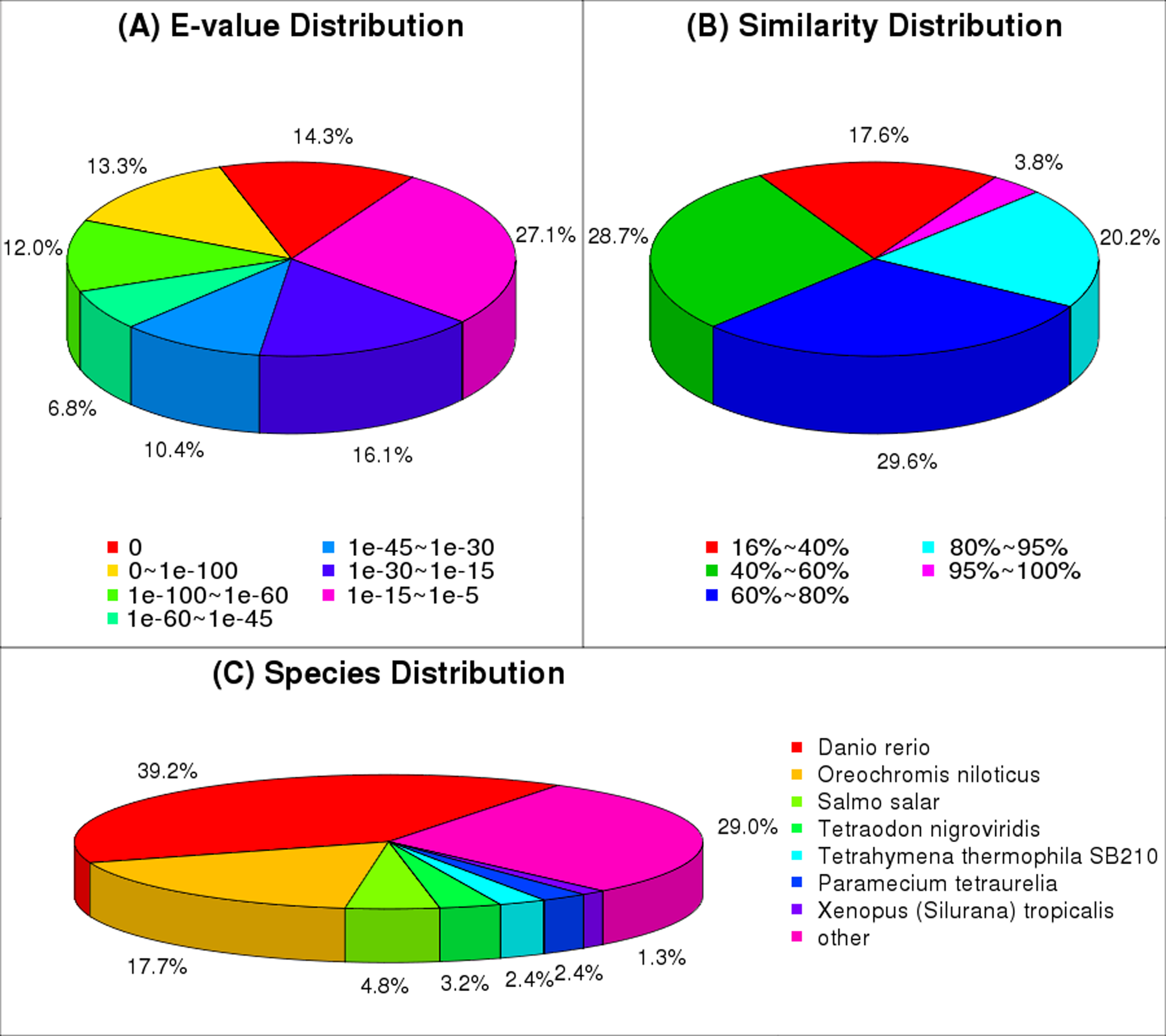

Supplement: Figure S3 — The NR database classification. (TIF) [file pone.0103832.s003.tif]

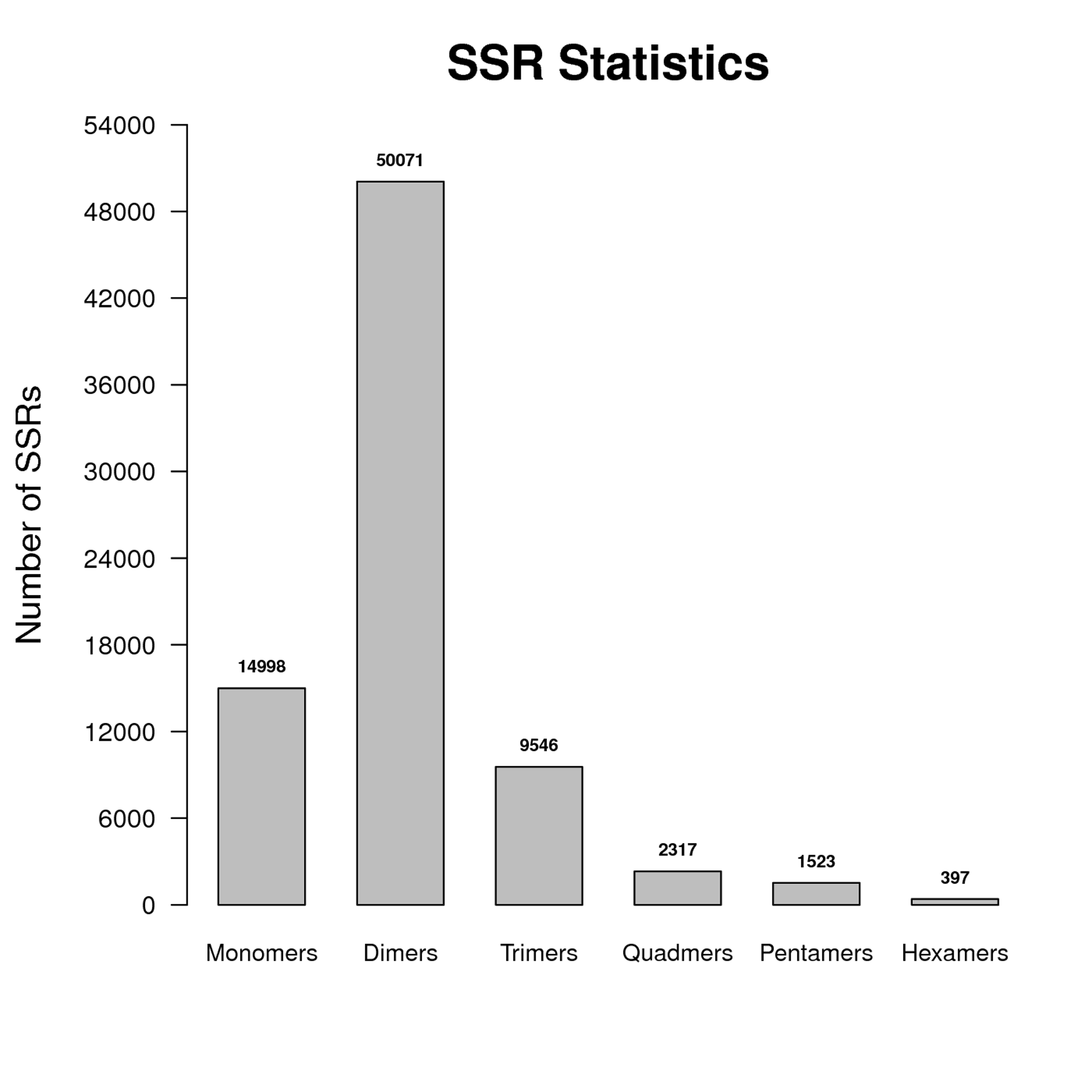

Supplement: Figure S4 — Simple sequence repeat statistics. (TIF) [file pone.0103832.s004.tif]
